# Cemented versus uncemented stems in total hip arthroplasty: No independent effect on transfusion or complications despite reduced total blood loss

**DOI:** 10.1002/jeo2.70734

**Published:** 2026-05-04

**Authors:** Nikolai Ramadanov, Dakota Fuchs, Maximilian Heinz, Robert Prill, Roland Becker

**Affiliations:** ^1^ Center of Orthopaedics and Traumatology, Brandenburg Medical School University Hospital Brandenburg an der Havel Brandenburg an der Havel Germany; ^2^ Faculty of Health Science Brandenburg Brandenburg Medical School Theodor Fontane Brandenburg an der Havel Germany

**Keywords:** blood loss, cemented stem, hidden blood loss, perioperative blood management, total hip arthroplasty, transfusion, uncemented stem

## Abstract

**Purpose:**

The impact of femoral stem fixation on perioperative blood loss and transfusion in total hip arthroplasty (THA) remains controversial. To compare cemented and uncemented stem fixation in elective primary THA regarding perioperative blood loss, transfusion requirements and postoperative complications. We hypothesized that stem fixation would not independently influence transfusion or complication risk after adjustment for patient‐related factors.

**Methods:**

This retrospective cohort study analysed data from a institutional registry including THAs performed between 2016 and 2023. Patients were stratified according to femoral stem fixation (cemented vs. uncemented). Outcomes included intraoperative, total and hidden blood loss (HBL), allogeneic red blood cell transfusion and postoperative complications. Multivariable linear and logistic regression analyses assessed the independent effect of stem fixation, adjusting for age, sex, body mass index, American Society of Anesthesiologists (ASA) class, operative time and preoperative haemoglobin.

**Results:**

A total of 648 patients were included (uncemented *n* = 548; cemented *n* = 100). Patients in the cemented group were older (79.8 ± 7.4 vs. 72.4 ± 9.1 years) and more frequently female (76.0% vs. 65.8%). Transfusion rates were higher in the cemented group. After multivariable adjustment, cemented fixation was independently associated with lower total blood loss (adjusted mean difference −146 mL; 95% confidence interval [CI] −271 to −21), whereas no independent associations were observed for intraoperative and HBL. Stem fixation was not independently associated with transfusion requirement (adjusted odds ratio [OR] 1.06; 95% CI 0.56–1.99) or postoperative complications (adjusted OR 0.64; 95% CI 0.21–1.98).

**Conclusion:**

In elective primary THA, cemented femoral stem fixation is associated with a modest reduction in total blood loss but does not independently influence transfusion rates or postoperative complication risk. Clinically, these findings indicate that fixation should not be selected based on expectations of reduced transfusion risk. Instead, perioperative blood management should focus on patient‐related factors, particularly preoperative haemoglobin optimization.

**Level of Evidence:**

Level III, retrospective comparative cohort study.

## Graphical abstract

Cemented versus uncemented stems in total hip arthroplasty: reduced total blood loss with cemented fixation, but similar transfusion and complication rates.

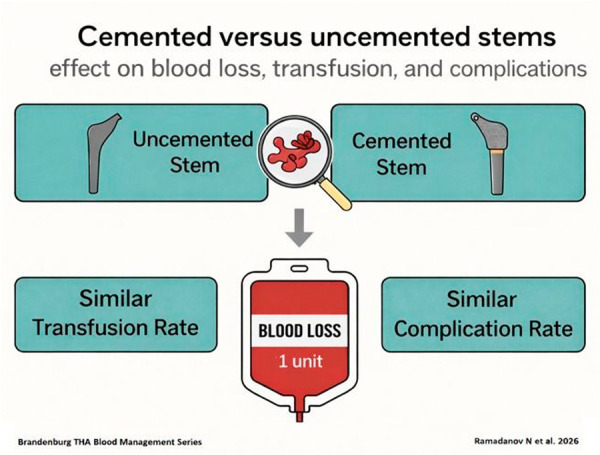

**Abbreviations**
ASA: American Society of Anesthesiologists
BMI: body mass index
CI: confidence interval
DVT: deep vein thrombosis
GT: greater trochanter
Hb: haemoglobin
HBL: hidden blood loss
Hct: haematocrit
MCH: mean corpuscular haemoglobin
MCHC: mean corpuscular haemoglobin concentration
MCV: mean corpuscular volume
MD: mean difference
OR: odds ratio
PE: pulmonary embolism
PMMA: polymethylmethacrylate
PRBC: packed red blood cells
RBC: red blood cell
RCT: randomized controlled trial
STROBE: Strengthening the Reporting of Observational Studies in Epidemiology
THA: total hip arthroplasty
WHO: World Health Organization

## INTRODUCTION

Total hip arthroplasty (THA) is one of the most successful procedures in orthopaedic surgery, offering durable pain relief, restored mobility and high patient satisfaction [10]. Both cemented and uncemented femoral stems achieve excellent long‐term survivorship; however, the optimal fixation method remains debated with regard to implant‐related complications and long‐term outcomes [2, 8]. The impact of fixation technique on perioperative blood loss and transfusion remains less clearly defined. Cemented stems provide immediate stability through polymethylmethacrylate (PMMA) interdigitation with cancellous bone, while uncemented stems rely on press‐fit fixation followed by osseointegration [12].

Comparative evidence has primarily focused on implant‐related complications and survivorship, with registry data and randomized controlled trials reporting higher rates of periprosthetic fractures after uncemented fixation and a reduced early fracture risk with cemented stems [1, 7, 9, 19]. Long‐term analyses likewise suggest differences in revision‐free survival between fixation methods [1, 2]. However, in contrast to these well‐studied outcomes, the impact of fixation technique on perioperative blood loss and transfusion remains less clearly defined and has received comparatively limited attention.

By contrast, the impact of fixation on perioperative blood loss and transfusion remains less consistent. Some studies report no significant difference, while others implicate stem geometry, with short or neck‐preserving designs associated with reduced blood loss compared to conventional implants [18]. Independent of fixation technique, preoperative haemoglobin has consistently emerged as the single strongest predictor of transfusion, with average rates of 15%–20% even in cohorts receiving modern tranexamic acid prophylaxis [4].

Despite extensive literature, important gaps remain. Many comparative studies are derived primarily from elderly populations undergoing hemiarthroplasty for fracture, limiting their applicability to elective THA. In addition, heterogeneity in methods of blood loss estimation, transfusion thresholds and implant designs hinders generalizability. Furthermore, the influence of contemporary perioperative blood management strategies on fixation‐specific outcomes has not yet been fully elucidated.

From a mechanistic perspective, cemented fixation may influence perioperative blood loss through intramedullary pressurization and partial sealing of the femoral canal, potentially reducing ongoing bleeding from cancellous bone surfaces. In contrast, uncemented press‐fit preparation may result in greater exposure of trabecular bone and intramedullary bleeding. However, these theoretical considerations have not been consistently confirmed in clinical studies.

Therefore, the aim of this retrospective analysis was to compare cemented and uncemented femoral stems in elective primary THA with respect to perioperative blood loss, transfusion requirements and complication rates. We hypothesized that cemented fixation may reduce perioperative blood loss by partially sealing the intramedullary canal, but that, after adjustment for patient‐related factors, stem fixation would not independently influence transfusion or complication risk.

## METHODS

### Ethics approval

This retrospective study was approved by the Ethics Committee of the University of Brandenburg (Reference No. 292032025‐BO‐E‐RETRO). The present work represents a predefined secondary analysis conducted under the scope of this original approval.

### Study design and setting

In accordance with the STROBE statement (see Data S3 for the completed checklist) [5], this retrospective study used data derived from a prospectively maintained institutional registry at the University Hospital Brandenburg/Havel, encompassing all elective primary THAs performed between 2016 and 2023. The present study is part of the *Brandenburg THA Blood Management Series*, a group of methodologically independent analyses based on the same underlying registry cohort [15, 16]. Each study addresses a distinct predefined research question with a separate exposure definition and analytical approach [15, 16].

#### Study population

All patients undergoing elective primary THA were considered eligible for inclusion. Patients were categorized based on femoral stem fixation into cemented and uncemented cohorts.

### Inclusion and exclusion criteria

Each patient was included only once and categorized according to femoral stem fixation (cemented vs. uncemented). Exclusion criteria were procedures performed for fracture‐related indications (including both pathological and non‐pathological fractures), hemiarthroplasty, conversion from prior osteosynthesis and revision arthroplasty. Acetabular fixation was not part of the exposure definition; cases involving isolated cup cementation were not specifically recorded and cannot be completely excluded, but are expected to be rare and unlikely to influence the results. Indications for surgery included primary osteoarthritis, avascular femoral head necrosis, dysplasia‐associated osteoarthritis and post‐traumatic osteoarthritis. All included procedures were elective primary THAs.

### Surgical technique

All surgeries were performed at a certified high‐volume EndoCert arthroplasty centre using standardized perioperative protocols. Elective primary procedures were consistently conducted via a modified anterolateral approach based on the technique described by Watson‐Jones [20]. Procedures were performed by or under the supervision of experienced arthroplasty surgeons. Multiple surgeons were involved; however, all followed standardized institutional protocols. Cemented fixation was selected based on patient‐specific factors, particularly advanced age and reduced bone quality, whereas uncemented fixation was primarily used in younger patients with adequate bone stock. Thus, fixation technique was not randomly assigned but determined according to clinical indication, which may explain baseline differences between groups. Cemented stems (TwinSys, Mathys) and uncemented stems (TwinSys or Optimys, Mathys) were used. Cementation was performed using Palacos R bone cement, mixed under vacuum, with a standardized third‐generation technique, including pulsatile lavage, use of a distal cement restrictor, retrograde cement application and pressurization. Particular attention was paid to achieving adequate stem fit within the femoral canal prior to cementation.

### Data collection and processing

Data extraction was performed independently by two investigators (N. R. and D. F.) using the hospital information system. Operative reports, anaesthesia records, discharge summaries and internal quality assurance documents were systematically reviewed. Missing values were handled by complete‐case analysis; no imputation was performed.

Extracted variables included a comprehensive set of demographic, clinical, surgical and laboratory parameters recorded in the institutional registry. For the present analysis, variables relevant to the predefined study objectives were selected, including age, sex, body mass index (BMI), American Society of Anesthesiologists (ASA) classification, preoperative haemoglobin, operative time and stem fixation. Additional variables (e.g., surgeon experience, cup inclination and detailed laboratory indices) were recorded as part of the registry and are analysed in separate studies within the same research framework, but were not included in the primary analyses of this manuscript. Transfusion decisions were made according to institutional clinical practice, based on haemoglobin levels in combination with patient symptoms and overall clinical status. A restrictive transfusion strategy was generally applied. Perioperative blood management followed standardized institutional protocols, including routine use of tranexamic acid and preoperative optimization where indicated; however, no formal patient blood management algorithm was implemented.

Patient age and BMI were calculated from the extracted data. Age was dichotomized using a threshold of 65 years, and BMI was categorized according to World Health Organization criteria. Discrepancies in data extraction were resolved by consensus between the two investigators.

Hidden blood loss was defined as the difference between calculated total blood loss and directly measured perioperative blood loss. Patient blood volume was estimated using the Nadler formula [14], and total blood loss was calculated using the Gross/Sehat dilution method [6, 17]. Measured blood loss comprised intraoperative suction volumes documented in the anaesthesia record and transfusion volumes. When exact transfusion volumes were unavailable, one unit of packed red blood cells (RBCs) was assumed to correspond to 300 mL.

Surgeon experience was defined based on the level of training. Procedures were primarily performed by certified senior arthroplasty surgeons (Hauptoperateure) at a high‐volume centre; in a minority of cases, procedures were performed by trainees under direct supervision. Operative time was defined as the duration from skin incision to wound closure, as recorded in the anaesthesia protocol. Cup inclination was obtained from routine postoperative anteroposterior pelvic radiographs as documented in institutional quality assurance records. Measurements were not reassessed for the purpose of this study.

### Variables and outcomes

Baseline variables included age, sex, BMI, ASA classification, preoperative haemoglobin, operative time and surgeon experience. The primary outcomes were perioperative blood loss, including intraoperative blood loss, total blood loss and hidden blood loss (HBL), allogeneic RBC transfusion, postoperative complications, including infection, dislocation, greater trochanter avulsion, periprosthetic fracture, reoperation, nerve injury, venous thromboembolism and death. A composite endpoint (‘any complication’) was additionally analysed.

### Statistical analysis

Continuous variables are reported as mean ± standard deviation and categorical variables as absolute counts and percentages. Group comparisons were performed using independent‐samples *t* tests or Fisher's exact test, as appropriate. To assess the independent effect of stem fixation on blood loss, multivariable linear regression models were applied, adjusting for age, sex, BMI, ASA class, operative time and preoperative haemoglobin. Robust standard errors were used. The association between stem fixation and transfusion, as well as postoperative complications, was evaluated using multivariable logistic regression, adjusting for the same covariates. Effect estimates are presented as adjusted mean differences (MDs) or odds ratios (ORs) with 95% confidence intervals (CIs). A two‐sided *p* value < 0.05 was considered statistically significant.

## RESULTS

A total of 648 patients undergoing elective primary THA were included, of whom 548 (84.6%) received an uncemented stem and 100 (15.4%) a cemented stem. Patients in the cemented group were older (79.8 ± 7.4 vs. 72.4 ± 9.1 years) and more frequently female (76.0% vs. 65.8%), with higher ASA classification (ASA III–IV: 82.0% vs. 59.6%) and lower preoperative haemoglobin (122.4 ± 14.9 vs. 128.6 ± 13.7 g/L) (Table 1). Operative time did not differ significantly between groups (86.9 ± 34.1 vs. 81.2 ± 29.6 min; *p* = 0.09). Surgeon experience was comparable between groups (95.4% vs. 96.0% senior surgeons; *p* = 1.00).

**Table 1:** Baseline characteristics and unadjusted outcomes of patients undergoing elective primary total hip arthroplasty stratified by stem fixation (cemented vs. uncemented).

Variable	Uncemented (*n* = 548)	Cemented (*n* = 100)	*p* value
Age (years)	72.4 ± 9.1	79.8 ± 7.4	<0.001
Female sex (%)	65.8	76.0	0.048
BMI (kg/m^2^)	28.3 ± 5.6	29.2 ± 5.9	0.18
ASA III–IV (%)	59.6	82.0	<0.001
Preoperative Hb (g/L)	128.6 ± 13.7	122.4 ± 14.9	<0.001
Operative time (min)	81.2 ± 29.6	86.9 ± 34.1	0.09
Any transfusion (%)	12.9	33.0	<0.001
Any complication (%)	3.8	7.0	0.18
Surgeon experience (senior, %)	95.4	96.0	1.00

Abbreviations: ASA, American Society of Anesthesiologists; BMI, body mass index; CI, confidence interval; Hb, haemoglobin.

**Figure 1:**
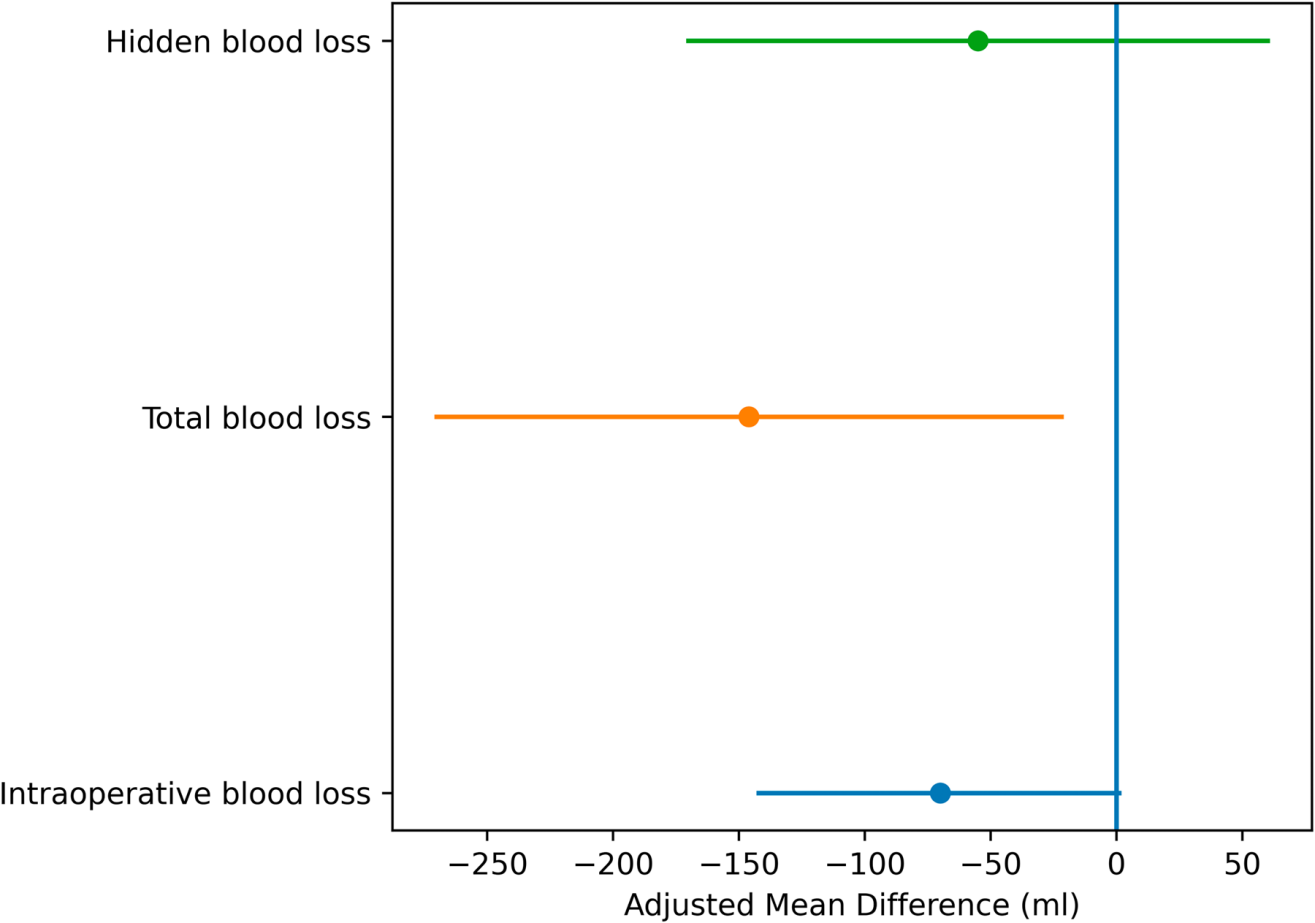
Adjusted mean differences in perioperative blood loss comparing cemented and uncemented femoral stems. Error bars represent 95% confidence intervals.

In unadjusted analyses, intraoperative blood loss was higher in uncemented compared with cemented THA, whereas no significant differences were observed for total or HBL (Table 2). After multivariable adjustment, cemented stem fixation was independently associated with lower total blood loss (adjusted MD −146 mL, 95% CI −271 to −21; *p* = 0.022), whereas no significant associations were observed for intraoperative blood loss (adjusted MD −70 mL, 95% CI −143 to 2; *p* = 0.057) or HBL (adjusted MD −55 mL, 95% CI −171 to 61; *p* = 0.351) (Table 2, Figure 1).

**Table 2:** Adjusted associations between stem fixation and perioperative blood loss (intraoperative, total and hidden blood loss).

Outcome	Adjusted mean difference (mL)	95% CI	*p* value
Intraoperative blood loss	−70	−143 to 2	0.057
Total blood loss	−146	−271 to −21	0.022
Hidden blood loss	−55	−171 to 61	0.351

*Note*: Multivariable models were adjusted for age, sex, BMI, ASA classification, operative time and preoperative haemoglobin.

Abbreviations: ASA, American Society of Anesthesiologists; BMI, body mass index; CI, confidence interval.

Transfusion rates were higher in the cemented group (33.0% vs. 12.9%; *p* < 0.001). However, after adjustment for age, sex, BMI, ASA class, operative time and preoperative haemoglobin, stem fixation was not independently associated with transfusion requirement (adjusted OR 1.06, 95% CI 0.56–1.99; *p* = 0.867) (Table 3, Figure S1).

**Table 3:** Unadjusted and adjusted associations between stem fixation and allogeneic red blood cell transfusion.

Analysis	Odds ratio	95% CI	*p* value
Unadjusted	3.32	2.04 to 5.40	<0.001
Adjusted	1.06	0.56 to 1.99	0.867

*Note*: Multivariable models were adjusted for age, sex, BMI, ASA classification, operative time and preoperative haemoglobin.

Abbreviations: ASA, American Society of Anesthesiologists; BMI, body mass index; CI, confidence interval.

Postoperative complication rates were as follows: infection 1.1% in the uncemented group versus 2.0% in the cemented group, dislocation 0.7% versus 1.0%, periprosthetic fracture 0.9% versus 3.0%, reoperation 2.2% versus 1.0% and mortality 0.0% versus 2.0%. After multivariable adjustment, cemented fixation was not associated with an increased risk of overall complications (OR 0.64; 95% CI 0.21–1.98), with similarly non‐significant results observed for individual complications (Table 4, Figure S2). Although rates were numerically higher in the cemented group for most outcomes, these differences were not statistically significant. Due to the low number of events, estimates for individual complications showed wide CIs.

**Table 4:** Unadjusted postoperative complication rates according to stem fixation.

Outcome	Uncemented (%)	Cemented (%)	*p* value
Infection	1.1	2.0	0.29
Dislocation	0.7	1.0	0.57
Periprosthetic fracture	0.9	3.0	0.11
Reoperation	2.2	1.0	0.70
Death	0.0	2.0	0.023

*Note*: Values represent unadjusted percentages; no adjusted analyses are presented in this table.

## DISCUSSION

Cemented femoral stem fixation was independently associated with a modest reduction in total blood loss after multivariable adjustment, whereas stem fixation did not independently influence transfusion rates or postoperative complication risk. These findings suggest that fixation technique itself has a limited role in determining clinically relevant blood management outcomes when patient‐related and perioperative factors are appropriately accounted for.

From a clinical perspective, this indicates that the choice of fixation method should not be primarily driven by concerns regarding blood loss or transfusion risk. Instead, patient‐specific factors, particularly preoperative haemoglobin and overall clinical status, appear to be more relevant determinants of perioperative blood management.

After adjustment for relevant confounders, cemented stems were associated with lower total blood loss, whereas no independent differences were observed for intraoperative or HBL. The magnitude of this effect was modest (−146 mL) and unlikely to translate into clinically relevant differences such as transfusion requirement. These findings support the concept that perioperative blood loss in THA is primarily driven by patient‐related and perioperative factors rather than implant fixation alone [3, 11, 15].

Although transfusion rates were substantially higher in the cemented cohort on unadjusted analysis, this association was no longer present after multivariable adjustment. This finding is clinically relevant, as it demonstrates that the observed difference in transfusion frequency is largely explained by confounding factors such as advanced age, higher ASA class and lower preoperative haemoglobin in patients receiving cemented stems. These results reinforce the concept that transfusion risk in elective THA is primarily patient‐driven rather than implant‐driven, and they are consistent with prior studies identifying preoperative haemoglobin as the strongest predictor of transfusion across fixation techniques.

Postoperative complications were infrequent in both groups, and no independent association between stem fixation and overall complication risk was observed after adjustment. While unadjusted analyses suggested numerically higher complication rates in the cemented group, these differences were not statistically significant and were attenuated after adjustment for baseline risk differences. Due to the low event rates, particularly for individual complications such as periprosthetic fracture or mortality, these findings should be interpreted cautiously. Nevertheless, the absence of a fixation‐dependent signal suggests that both cemented and uncemented stems can be used without relevant differences in blood loss, transfusion or complication rates when appropriate patient selection is applied.

The absence of a difference in operative time despite a higher‐risk profile in the cemented group may partly be explained by surgeon‐related factors. Importantly, surgeon experience was comparable between groups, suggesting that confounding by surgeon experience is unlikely.

The present findings should be interpreted in the context of existing literature. Much of the comparative evidence on cemented versus uncemented fixation is derived from fracture‐related arthroplasty or elderly populations undergoing hemiarthroplasty, limiting its applicability to elective THA. However, recent data in elective THA have reported lower blood loss with cemented or hybrid fixation, consistent with our findings of reduced total blood loss after adjustment [13]. Importantly, our analysis extends these observations by demonstrating that this effect does not translate into differences in transfusion requirement or complication rates, highlighting the limited clinical impact of fixation technique on perioperative blood management. By focusing exclusively on elective primary THA and incorporating contemporary perioperative blood management practices, the present study provides a more targeted assessment of fixation‐specific effects on blood loss and transfusion. The results of this study complement registry‐based survivorship and complication data by demonstrating that fixation technique alone does not substantially alter perioperative haematologic outcomes.

From a clinical perspective, these results have practical implications for perioperative blood management. The lack of an independent association between stem fixation and transfusion suggests that efforts to reduce transfusion rates should focus on preoperative optimization, particularly correction of anaemia, rather than implant selection. Similarly, the small reduction in total blood loss associated with cemented fixation should not be overinterpreted in the context of perioperative blood management, as fixation choice remains primarily guided by patient age, bone quality and fracture risk rather than expectations of reduced bleeding.

Several limitations should be acknowledged. First, the retrospective design introduces the potential for residual confounding despite multivariable adjustment. Second, the study was conducted at a single high‐volume centre with standardized protocols, which may limit generalizability. Third, although the overall cohort was large, event rates for individual complications were low, restricting the precision of risk estimates. Fourth, transfusion thresholds and perioperative management evolved over the long study period, which may have influenced absolute transfusion rates. Fifth, the proportion of cemented cases was substantially lower than that of uncemented cases, reflecting real‐world clinical practice but introducing potential imbalance between groups. Although multivariable adjustment was applied to account for confounding, residual confounding cannot be excluded. Sixth, no matching or propensity score techniques were used. Instead, we chose a regression‐based approach to preserve the full sample size and maintain generalizability of the findings.

## CONCLUSION

In elective primary THA, cemented femoral stem fixation is associated with a small reduction in total blood loss but does not independently affect transfusion requirements or postoperative complication rates. These findings indicate that stem fixation plays a limited role in perioperative blood management compared with patient‐related factors and underscore the importance of individualized, evidence‐based decision‐making in elective THA.

## AUTHOR CONTRIBUTIONS

**Nikolai Ramadanov:** Conceptualization; data curation; formal analysis; investigation; methodology; project administration; resources; software; supervision; validation; visualization; writing—original draft; writing—review and editing. **Dakota Fuchs:** Data curation; formal analysis; investigation. **Maximilian Heinz:** Data curation; formal analysis; investigation. **Robert Prill:** Writing—review and editing. **Roland Becker:** Writing—review and editing.

## CONFLICT OF INTEREST STATEMENT

The authors declare no conflicts of interest.

## ETHICS STATEMENT

This retrospective study was approved by the Ethics Committee of the University of Brandenburg (Reference No. 292032025‐BO‐E‐RETRO). All procedures were conducted in accordance with institutional regulations and the Declaration of Helsinki (latest revision). Because the study used fully anonymised, routinely collected clinical data without direct patient contact, the Ethics Committee formally waived the requirement for obtaining individual informed consent.

## Supporting information

Supplementary Figure 1. Adjusted odds ratios for allogeneic blood transfusion comparing cemented versus uncemented stem fixation.

Supplementary Figure 2. Adjusted odds ratio for postoperative complications comparing cemented versus uncemented femoral stem fixation. The estimate refers to the composite endpoint “any complication” and was derived from multivariable logistic regression adjusted for age, sex, BMI, ASA class, operative time, and preoperative hemoglobin. Error bars indicate 95% confidence intervals.

STROBE‐checklist‐v4‐cohort.

## Data Availability

The data extraction set is available from the corresponding author upon reasonable request.
