# Supplementary figures and images for: Cemented versus uncemented stems in total hip arthroplasty: No independent effect on transfusion or complications despite reduced total blood loss
Source: J Exp Orthop. 2026 May 4;13(2):e70734. doi: 10.1002/jeo2.70734 (PMC13137437; doi:10.1002/jeo2.70734)

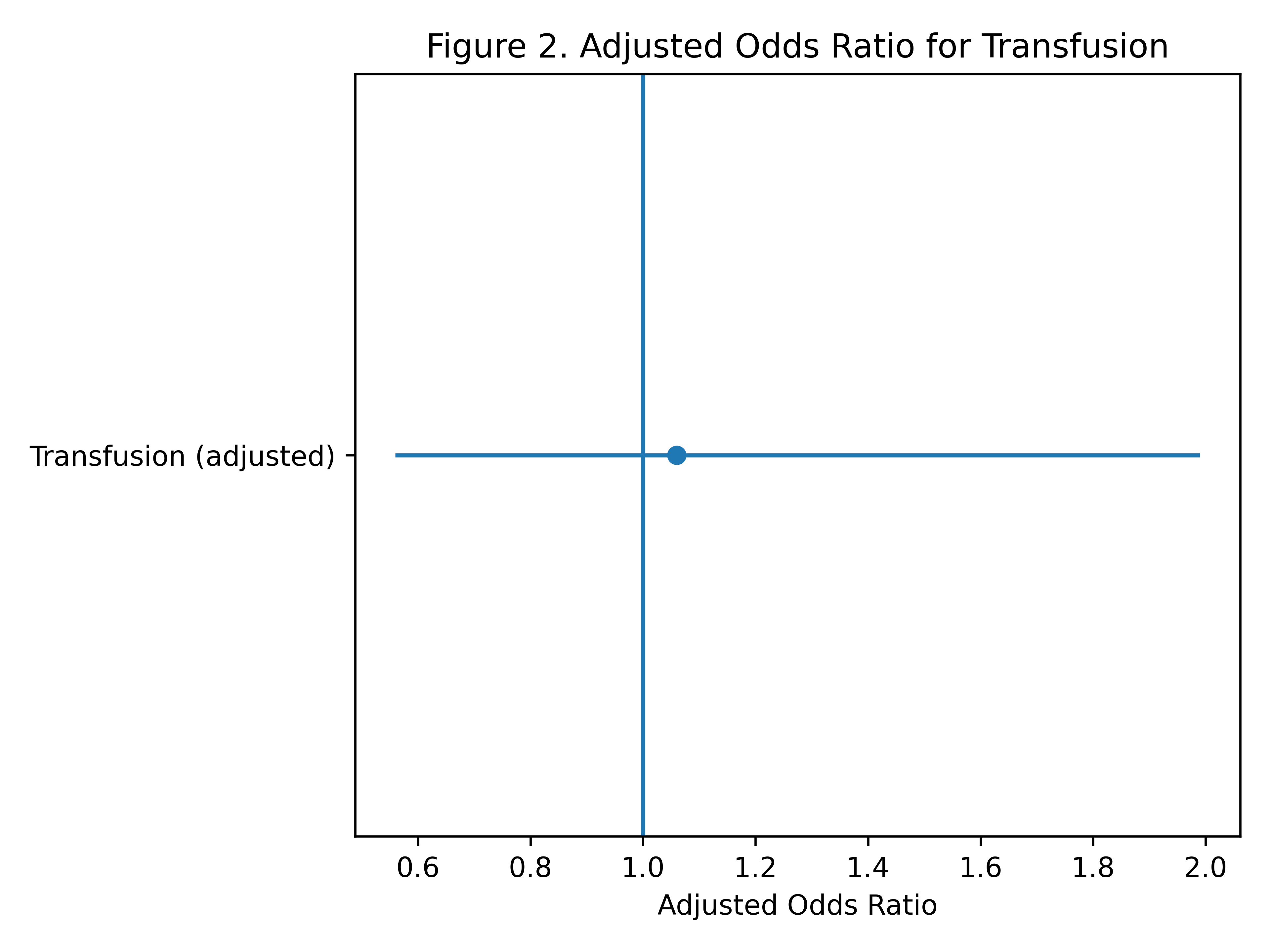

Supplement: Supplementary file 1 — Supplementary Figure 1. Adjusted odds ratios for allogeneic blood transfusion comparing cemented versus uncemented stem fixation. [file JEO2-13-e70734-s002.png]

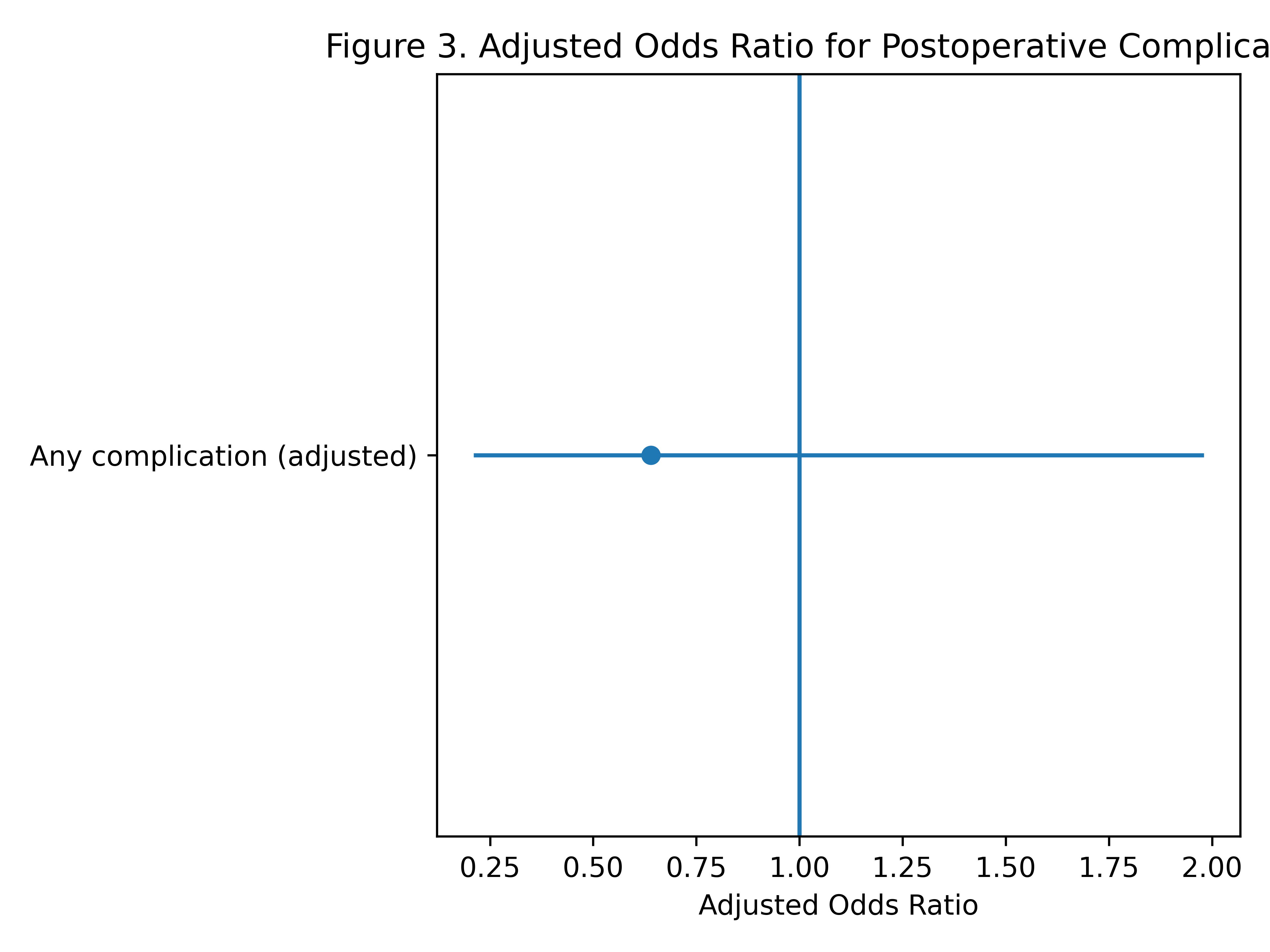

Supplement: Supplementary file 2 — Supplementary Figure 2. Adjusted odds ratio for postoperative complications comparing cemented versus uncemented femoral stem fixation. The estimate refers to the composite endpoint “any complication” and was derived from multivariable logistic regression adjusted for age, sex, BMI, ASA class, operative time, and preoperative hemoglobin. Error bars indicate 95% confidence intervals. [file JEO2-13-e70734-s001.png]
